# Epigenetic Dynamics: Role of Epimarks and Underlying Machinery in Plants Exposed to Abiotic Stress

**DOI:** 10.1155/2014/187146

**Published:** 2014-09-18

**Authors:** Manoj Kumar Dhar, Parivartan Vishal, Rahul Sharma, Sanjana Kaul

**Affiliations:** ^1^Plant Genomics Laboratory, School of Biotechnology, University of Jammu, Jammu 180006, India; ^2^School of Biotechnology, University of Jammu, Jammu 180006, India

## Abstract

Abiotic stress induces several changes in plants at physiological and molecular level. Plants have evolved regulatory mechanisms guided towards establishment of stress tolerance in which epigenetic modifications play a pivotal role. We provide examples of gene expression changes that are brought about by conversion of active chromatin to silent heterochromatin and vice versa. Methylation of CG sites and specific modification of histone tail determine whether a particular locus is transcriptionally active or silent. We present a lucid review of epigenetic machinery and epigenetic alterations involving DNA methylation, histone tail modifications, chromatin remodeling, and RNA directed epigenetic changes.

## 1. Introduction

Stress is inevitable in the life cycle of living organisms. Being sessile, plants are more prone to the deleterious effects of environmental stress. Depending upon whether the factors involved are living or nonliving, environmental stress can be categorized as biotic (plant pathogens, etc.) or abiotic stress (drought, salinity, chilling, etc.). Stressful conditions generally do not occur as isolated events but as crosstalk of multiple stresses. Therefore, plants have developed complex mechanisms to survive under these challenging conditions. Tolerance, avoidance, and resistance are three major strategies followed by plants to counter the recurring biotic and abiotic stresses. These mechanisms involve genes associated with several interconnected pathways which lead them towards better stress tolerance [1]. Plants resort to various modifications in their morphological traits, physiology, and so forth in response to stress.

Depending upon whether the stress is permanent or transitory, plants respond through various short term as well as long term strategies. Short term strategies include alteration in the plant homeostasis. Restoration of cellular homeostasis reduces stress injury by eliminating consequences of stress which leads to development of stress tolerance. Long-term strategies include transgenerational changes involving development of heritable gene expression changes. This comprises creation of new epigenetic marks while erasing old ones and increasing the expression of some genes while silencing some other genes. Severe and prolonged stress can lead to genome alterations which may sometimes contribute towards better adaptation [2]. The basic information guiding the behavior of plant lies in the DNA sequence and alterations in DNA sequence by mutation or genetic recombination lead to new alleles which may confer enhanced stress tolerance to the plant. However, the rate of formation of new gene combinations is too slow in comparison to the occurrence of different stresses in the environment [3]. Therefore, the survival of plant in these conditions depends largely on the regulation of various stress responsive genes, that is, epigenetic mechanisms. Epigenetic changes include any heritable change in an organism which does not involve change in the DNA sequence. Rather, it involves addition or deletion of epimarks (methylation, etc.) on DNA, posttranslational modifications on histone tails (acetylation, methylation, sumoylation, etc.), and RNA interference.

Histone modifications and alterations in DNA methylation are together referred to as epigenetic regulation but only those changes which are either mitotically or meiotically heritable are truly epigenetic [4]. These alterations affect gene expression by chromatin remodeling which involves change in chromatin state of the chromosome, that is, euchromatin or heterochromatin. For instance, in order to increase the expression of a particular gene, its promoter sequence must be exposed so that transcription factor and RNA polymerase could bind to the underlying upstream DNA and carryout transcription of the gene. In order to expose the DNA for efficient transcription, nucleosome complex must be untangled. Conversely, for shutting off gene expression, DNA methylation has to be reestablished, followed by packaging of the DNA stretch covering that particular gene by the histone components of the nucleosome complex.

## 2. Epigenetic Alterations and Requisite Machinery

### 2.1. DNA Methylation

Methylation is the only covalent modification that has been identified on DNA till date [5]. It involves addition of methyl group (−CH_3_) at fifth carbon in the cytosine ring of the DNA molecule at CpG, CpNpG (symmetric), or CpNpN (asymmetric) sites (where N is A, C, or T).

Mechanism of DNA methylation is governed by two types of enzymes:

- methyltransferases;
- demethylases.

Two major enzymatic activities regulate cytosine methylation in plants which involve* de novo* establishment of methylation on DNA and maintenance of the already methylated DNA. The* de novo* methylation is a process by which previously unmethylated cytosine residues are methylated, leading to the formation of new methylation patterns. Maintenance methylation is the process of maintenance of preexisting methylation patterns after DNA replication [6]. MET1 (DNA methyltransferase 1) and CMT (chromomethylase) are responsible for maintenance of CG and CNG methylation, respectively [7].* De novo* methylation is established by DRM2 (domains rearranged methyltransferase 2) in the new DNA sequences generated after DNA replication. DNA gycosylases (ROS1, DML2, DML3, and DME) catalyze the removal of methyl group from cytosine residue [8].

Genome-wide analysis of DNA methylation in* Arabidopsis thaliana* revealed the methylation status of its genome as 24% CG, 6.7% CNG, and 1.7% CNN methylation [9]. CpNpG and CpNpN methylation changes mediated by CMT3 and DRM2 have been reported to regulate transposons and repeat regions through chromatin remodelling during exposure to stress [10, 11].

DNA methylation is distributed in the plant genome including heterochromatic and euchromatic regions [12]. The heterochromatic regions, densely packed with transposable elements and other repetitive sequences, are highly methylated whereas euchromatic regions, containing genes and nonrepetitive intergenic regions, show comparatively lesser cytosine methylation. Interestingly, transposons are methylated along their entire length in contrast to genes which are often methylated away from the start and termination sites. Within the euchromatic region, pseudogenes and transcriptionally inactive genes show higher levels of methylation than actively expressing genes [13]. Expressed genes are methylated in the transcribed region (gene-body methylation) [14]. Gene body methylation exhibits a parabolic relationship with transcription level in rice and* Arabidopsis*. Both the least expressed and the highly expressed genes are least prone to methylation whereas moderately expressed genes are most likely to be methylated at gene body [15, 16]. Genic regions do not contain non-CG methylation while transposons and repeats abundantly possess CpNpG or CpNpN methylation. Methylation at 5′ portion (promoter plus some transcribed region) and 3′ portion inhibits gene expression.

Stress can cause hypermethylation or hypomethylation of DNA. In maize roots, cold induced expression of ZmMI1 was accompanied with a decrease in DNA methylation which did not revert to basal level even after 7 days of recovery. In tobacco, aluminium, salt, cold, and paraquat stresses induced DNA demethylation at CG nucleotides in the coding sequence of NtGPDL gene (glycerophosphodiesterase-like protein) [17]. Heavy metal stress is known to induce hypomethylation at specific sites in the genome of both the metal-sensitive* Trifolium repens *L. and metal-tolerant* Cannabis sativa *L. [18]. DNA hypermethylation at CG but not CNG at two heterochromatic loci was induced in cell suspension culture of tobacco by osmotic stress [19]. Drought-induced hypermethylation has been proposed to play a primary and direct role in reducing the metabolic activity in pea root tips after 72-hour water deficit [20, 21]. Suji and Joel [22] reported drought induced hypermethylation and hypomethylation in drought tolerant and drought susceptible varieties of rice, respectively. Stress induced hypermethylation of satellite DNA was associated with a switch in photosynthesis mode from C3 to CAM in* Mesembryanthemum crystallinum* L., a facultative halophyte [23].

Promoter demethylation is known to abolish constitutive gene silencing established because of hypermethylation of* Xa21G* gene, thereby conferring disease resistance in rice [24]. Changed methylation level in maize exposed to osmotic and salt stress helps in stress acclimation [25]. Stressful environment produces transgenerational epigenetic modifications leading to enhanced stress adaptability in future progenies [26]. Nonstressed progenies of stressed rice plants carrying modified methylation patterns acquired from the parent exhibit enhanced stress tolerance [27].

Transposons and other repeats constitute large part of the plant genome and cytosine methylation is chiefly targeted towards transposon silencing [28, 29]. CG and non-CG methylation contribute towards transposon immobilization. In plants, non-CG methylation is proposed to have evolved as an epigenetic tag committed to transposon control [30]. A close relationship between methylation and low temperature dependent transposition (LTDT) has been reported, where low temperature caused reduction in methylation level opposed to hypermethylation resulting from higher temperature in* Antirrhinum majus* [31]. Transposon methylation changes which control transposition activity of transposons are also reported to spread silencing signal to neighboring genes.* Tos17* methylation spreads to upstream ABC-transporter-like gene [32].

### 2.2. Histone Code

Histones are very crucial for packaging of DNA. DNA folds around histone octamer (H2A, H2B, H3, and H4) to form nucleosome, which is the basic unit of chromatin. The organization of chromatin is critical for transcription and many other cellular processes like replication, repair, recombination, and so forth. This organization is directly influenced by posttranslational modifications in the histone tails protruding out of their amino terminal. The histone tails are reported to interact with negative charge on the DNA and other associated proteins [33]. These interactions are altered by certain posttranslational modifications targeted towards specific amino acid residues and depending upon the posttranslational modification on histone tail, the integrity of nucleosome in that region is determined. These modifications include methylation, acetylation, phosphorylation, ubiquitination, biotinylation, and sumoylation at specific amino acid residues [34]. A combination of site-specific posttranslational modifications on different residues of histone tail constitutes “histone code.” Each modification signifies a particular chromatin state and regulates transcriptional activity in combination with different external and internal signals.

### 2.3. Modifying Enzymes

Histone acetyltransferases (HATs) carry out acetylation of histone tails and are associated with gene activation. HATs transfer acetyl group to *ɛ*-amino group of lysine residues in the N-terminal extensions of nucleosomal core histones.

Lysine (K) bears positive charge and the transfer of acetyl group neutralizes this positive charge. This reduces the affinity of nucleosome complex for DNA leading to relaxed chromatin state and subsequent transcriptional activation. About 15 HATs have been reported in* Arabidopsis*, which belong to three families: GNAT/MYST, CBP, and TF II250 [35]. HATs interact with TFs and activate stress responsive genes which regulate stress tolerance. SAGA (HAT) interacts with ADA1 (TF) and the SAGA/ADA1 complex interacts with CBF1 which recruits this complex to activate downstream genes for better cold tolerance [36].

Histone deacetylases (HDACs) are responsible for deacetylation, that is, removal of acetyl group from histones, leading to condensed chromatin state and thereby causing gene silencing [37]. HDACs are further divided into three families [38], namely, (a) RPD3 family, (b) SIR2 family, and (c) HD2 family. Both HATs and HDACs affect the expression of developmental and stress responsive genes.

HMTs (histone methyl transferases) and HDMs (histone demethylases) are responsible for methylation and demethylation of histone tails, respectively.

Histone methylation occurs at lysine and arginine amino acids. All lysine methylations are carried out by HKMTs (histone lysine methyltransferases) containing SET domain [39]. They are classified in to five classes, Class I to Class V (Table 1).

*Histone Demethylases (HDMs)*. There are two types of demethylases which carry out oxidative demethylation of histones (Table 2).

### 2.4. Histone Modifications

#### 2.4.1. H3K Acetylation

**Figure 1:**
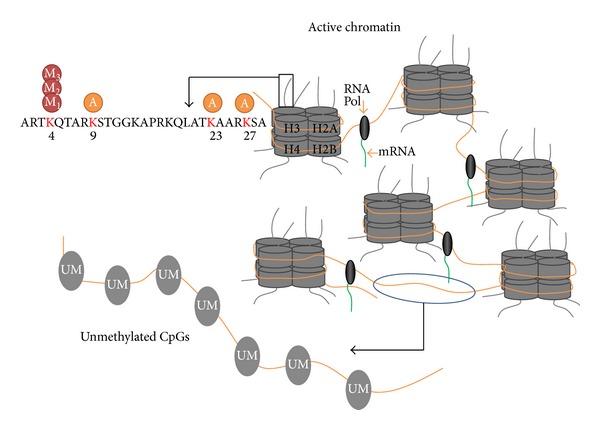
Epigenetic marks associated with transcriptionally active chromatin. Trimethylation at K4 and acetylation at K9, K23, and K27 of H3 and unmethylated CGs signify active chromatin.

Acetylation of lysine residues is very flexible and plays a vital role in the life cycle of plants [7]. Active chromatin is marked by H3 acetylation resulting in relaxation of chromatin state which facilitates the transit of RNA polymerase [42] (Figure 1). Histone lysine acetylation rearrangement has been reported to be associated with flowering [43] and cold stress tolerance [44]. HDA6 and HDA19 expression is induced by stress and affects local chromatin structure. HDA6 has been reported to be responsible for deacetylation of histones in response to biotic and abiotic stress induced by jasmonic acid and ethylene in* Arabidopsis.* Overexpression of AtHD2C in transgenic* Arabidopsis* results in increased expression of ABA-responsive genes (LEA) leading to improved salt and drought stress tolerance [45]. Hos15 protein interacts with H4 and carries out H4 deacetylation thereby regulating stress tolerance in* Arabidopsis* [44].

H3K4me3 and H3K9 acetylation on promoter region and H3K23 acetylation and H3K27 acetylation on coding region affect gene expression of stress responsive genes [35]. Four drought responsive genes (RD29A, RD29B, RD20, and RAP2.4) have been reported to exhibit enrichment of H3K4me3 and H3K9 acetylation and activation in response to drought stress. Moreover, there is a gradual decrease of nucleosomal density on RD20 and RAP2.4 genes under drought stress [35].

#### 2.4.2. H3K Methylation

**Figure 2:**
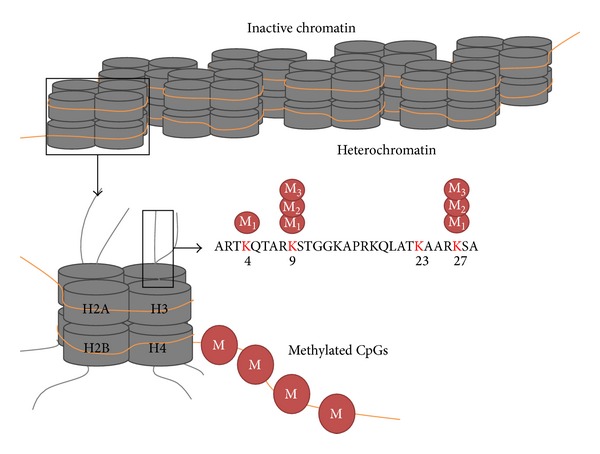
Epigenetic marks associated with transcriptionally inactive chromatin. Methylation at K4, K9, and K27 of H3 and methylated CGs indicate silent chromatin.

H3 lysine methylation is the most abundant histone modification. Lysine can be mono-, di-, or trimethylated. H3K9 methylation is a characteristic of heterochromatin and signifies silencing of the locus [46] (Figure 2). Despite this, the loss of this mark does not always represent the activation of the region suggesting the involvement of other important factors also [47]. H3K27me3 is a major chromatin silencing modification found associated with 5′ region of thousands of genes in* Arabidopsis* [48]. On the other hand, H3K9me3 is a repressive chromatin modification associated with gene coding region [49]. H3K9me2 is localized in heterochromatic region, transposons, pseudogenes, and repeats [50]. All H3K4me marks are associated with active chromatin. H3K4me3 and H3K4me2 are associated with promoter and 5′ part of the transcribed gene while H3K4me1 covers terminal part (3′) of the gene [51]. Silent chromatin (heterochromatin) bears H3K9me which recruits other proteins such as heterochromatin protein 1 (LHP1). These bind to methylated H3K9 and help in the propagation of heterochromatin to adjacent region of the chromosome [52].

Drought-inducible linker histone variant (H1S) in tomato is responsible for negative regulation of stomatal closure [53]. H3K4me3 and H3 acetylation was found to be induced in alcohol dehydrogenase 1 (ADH1) and pyruvate decarboxylase 1 (PDC1) genes. These changes were reverted back on withdrawal of submergence stress [54].

H3K4me marks on nucleosomes of stress-inducible genes have been reported to be associated with the activation of chromatin in response to dehydration [55]. H3Kme marks are reported to be present in 90% of annotated* Arabidopsis* genes wherein abundance of H3K4me3 mark is directly related to level of transcriptional activity of the drought responsive genes. Increase in H3 phosphorylation and H3 and H4 acetylation in response to abiotic stresses have been found in tobacco and* Arabidopsis* [56].

#### 2.4.3. Other Histone Modifications

In* Arabidopsis *an arginine methyltransferase SKB1 (also known as protein arginine methyl transferase5 PRMT5) is involved in abiotic stress response. SKB1 is normally associated with chromatin and increases level of arginine trimethylation on H4 (H4Rme2) so as to repress gene expression. With the onset of salt stress, SKB1 dissociates from chromatin and results in induction of stress responsive genes.* skb1* mutant is hypersensitive to salt stress [57].

### 2.5. RNA Directed DNA Methylation (RdDM)

Abiotic stress has been reported to modulate the expression of several hundred genes, and depending upon their roles they are either upregulated or downregulated. Apart from the regulatory control at the level of transcription, the posttranscriptional regulation is also important for regulation of gene expression. This is achieved by RNA binding proteins (RBPs) which bind to UTRs of mRNAs and control their stability, localization, or translation. In addition to this, small RNAs (microRNAs and small interfering RNAs) play a vital role in gene regulation [58]. RNAi machinery is necessary for the maintenance of heterochromatin and silencing of repetitive DNA, transposons, and so forth [59]. RNA directed DNA methylation (RdDM) is known to be regulated by temperature. Virus-induced gene silencing is promoted at low temperature and delayed by high temperature [60]. Though promoters are also methylated* de novo*, TEs and other repetitive DNA elements are effectively silenced by this mechanism [61].

#### 2.5.1. miRNA

MicroRNAs are short (20–24 nucleotide), endogenous RNAs, processed by Dicer-like enzyme from longer transcripts, which are not translated into proteins [58]. Plant miRNAs genes have been found away from protein coding regions of the genome and are expressed by their own transcription unit [62].

Role of miRNAs in gene regulation vis-à-vis abiotic stress has been best studied by Sunkar et al. [58]. Genes which are negative regulators of stress tolerance (i.e., repress stress responsive genes) are downregulated during stress by upregulation of microRNAs targeting these genes. On the other hand, miRNA downregulation under stress results in accumulation of mRNAs of those genes which act as positive regulators of stress tolerance [58].

Overexpression of miR396 in* Arabidopsis* and rice plants resulted in reduced tolerance to salt and alkali stress [63]. Sequence analysis of small RNA library of stress treated* Arabidopsis thaliana* showed that miR393 was the most abundantly expressed miRNA and its level increased by a variety of stresses like cold, salt, ABA, and dehydration. Some stress-specific expression of miRNAs was also observed; for instance, miR319c is upregulated by cold but not by ABA salt or dehydration [64]. Cold stress resulted in differential expression of a number of miRNAs including miR319 in rice and* Brachypodium* [65, 66]. On oxidative stress, miR398 is transcriptionally downregulated, therefore, leading to the accumulation of CSD1 and CSD2 mRNAs which are crucial for plant stress resistance. mRNAs of these two genes do not accumulate under normal conditions because of miR398-guided cleavage [67]. miR160 and miR164 along with their target genes have been reported to play an important role in the regulation of root growth in* Arabidopsis* during drought stress. Overexpression of miR160 led to agravitropic roots and increase in the number of lateral roots [68]. Manipulation of miRNA-guided gene regulation can help development of stress-resistant plants [69].

#### 2.5.2. siRNA

Small interfering RNAs (siRNAs), 20–24 nucleotides in length, are known to play an important role in a range of processes, such as heterochromatin formation, transposon silencing, transgene silencing, posttranscriptional regulation of mRNAs, and defense against viruses. Processing of long dsRNAs generated from natural cis-antisense gene pairs, repetitive DNA, or noncoding transcripts by Dicer-like enzymes generate small interfering RNAs [58].

After processing, one of the strands of the duplex serves as guide strand and is loaded onto RITS (RNA-induced transcriptional silencing complex). The complex binds to siRNA by PAZ domain of AGO4 protein and is directed to the homologous DNA sequence for gene silencing at transcriptional level (TGS). AGO4 is associated with Pol V which synthesizes transcripts that interact with siRNAs to induce DNA methylation at the targeted site by DRM2 (*de novo* methyltransferase) [10]. Nascent RNAs bind to the target DNA sequences and recruit histone methylases to add methyl group to lysine residues at 9 or 27 position of H3 histone tails. This leads to recruitment of DNA methylases which transfer methyl group to DNA ultimately leading to gene silencing and heterochromatin formation [70, 71]. The methylated DNA serves as template for Pol IV. Pol IV transcribes the methylated DNA and its downstream sequence to produce aberrant RNA transcripts which subsequently generates dsRNA by the activity RDR2 (RNA-dependant RNA polymerase 2). These RDR2 synthesized dsRNAs act as precursor for secondary siRNA which help in spreading methylation to adjacent sequences [10].

One of the possible mechanisms of regulation of plant stress response is the inhibition of siRNA biogenesis.* Dcl2* and* Dcl3* mutants having weakened transactivation activity of siRNA biogenesis were more sensitive to MMS (methyl-methane sulfonate) which causes genotoxic stress [72].

An excellent example of regulation of stress tolerance is that of genes involved in proline catabolism in* Arabidopsis*. SR05 is induced by salt stress. SR05 mRNA is complementary to P5CDH mRNA (P5CDH protein is an important enzyme for proline breakdown) and they together generate a dsRNA which is acted upon by siRNA biogenesis pathway factors (DCL2, RDR6, SGS3, and NRPD1A) to produce 24nt-siRNA. This nat-siRNA guides the cleavage of P5CDH mRNAs leading to proline accumulation and better salt tolerance. SR05 mutants exhibit hypersensitivity to salt stress [73].

### 2.6. Chromatin Remodelling Factors (CRMs)

Chromatin remodeling factors are multisubunit protein complexes which modify chromatin structure by influencing histone-DNA interactions in order to assemble, destabilize, or displace nucleosomes using ATP derived energy [74]. High CRM concentration results in histone octamer transfer to another DNA molecule. At moderate concentration, they facilitate sliding of the octamer position leading to altered gap between adjacent nucleosomes to facilitate access of TFs, restriction enzymes, and so forth.

ATP dependent chromatin remodeling factors can be grouped into three categories:

1. SWF/SNF ATPases;
2. ISWI (Imitation Switch) ATPases;
3. CHD (chromodomain and helicase-like domain) ATPases.

SWF1/SNF complex was originally identified for defects in mating type switching (SW1) and sucrose fermentation (sucrose nonfermenting) [75]. ATCHR12, a SNF2/Brahma-type chromatin-remodelling protein, plays an important role in temporary growth arrest of normally active primary buds in* Arabidopsis thaliana* exposed to stress [76]. SW13 subunit of SW1/SNF complex has been recently reported to act as a positive regulator in ABA-mediated inhibition of seed germination and growth by interacting with a negative regulator, HAB1 (Hypersensitive to ABA1), to increase the expression of RAB18 and RD29B [77]. Another chromatin remodeling factor PICKLE (PKL) helps in maintaining AB13 and AB15 chromatin in a repressed state during germination indicated by reduced H3K9 and H3K27 methylation level in* pkl *mutant seeds when treated with ABA [78].

Histone chaperons are known to carry out nucleosome assembly and disassembly by deposition or expulsion of histones, respectively. NAP1 (nucleosome assembly protein 1) is known to function as chaperon for H2A and H2B histones in* Arabidopsis* [79]. AtNAPs are reported to be positive regulators of ABA signaling pathway [80]. MSI1, a WD40 repeat protein acting as a subunit for many protein complexes (like chromatin assembly factor 1 and Polycomb group protein complexes), is involved in chromatin assembly and plays the role of a negative regulator in drought stress response in* Arabidopsis* [81]. Plants with highly reduced MSI1 levels exhibit enhanced level of ABA-responsive gene transcripts [82]. COR (Cold regulated) genes containing C/DRE (C-repeat/dehydration responsive element) are also regulated negatively by MSI1-like protein MSI4/FVE [83].

## 3. Conclusion

Stress-induced epigenetic changes in the form of DNA methylation, histone tail modifications, and RNA directed DNA methylation are governed by a complex phenomenon involving myriad factors interacting among themselves. These changes in epigenetic marks modulate transcription of stress-responsive genes leading to the formation of heritable epialleles which subsequently enable plant to withstand stress. There is a need for the identification of such epialleles along with comprehensive understanding of the fundamental epigenetic mechanisms. Importantly, it is necessary to study epigenetic heterogeneity (a key aspect of epigenetic dynamics) both at epialleles level and whole genome level [84]. Complete knowledge of these mechanisms would lay a platform for the researchers to devise better strategies for crop improvement like exploitation of small RNAs for the manipulation of epialleles.

## Figures and Tables

**Table 1:** Different classes of histone methyl transferases (HMTs).

	Class I	Class II	Class III	Class IV	Class V	
					SUVH	SUVR
Members	These are homologues of E(Z) (enhancer of zeste from *Drosophila*); three E(Z)-like proteins are encoded by *Arabidopsis *genome; CURLY LEAF (CLF) MEDEA (MEA) SWINGER (SWN)	This class is constituted by SDG8, SDG4	This class contains homologues of Trithorax; therefore, members are called ATX (*Arabidopsis* Trithorax-like) proteins 1–5 Five genes encode *Trithorax* homologues, ATX 1–5	*Arabidopsis *has two members belonging to this class, ATXR5 and ATXR6	SUVH (SU(VAR 3-9)) Ten plant specific members	[SU(VAR) 3-9 related]

Domains	Contains SET (suppressor of variegation, enhancer of zeste and Trithorax) domain, E(Z) domain, SANT domain (SW13, ADA2, NCOR, and TFIIIB-binding), and CXC (cysteine rich region)	Contain SET domain and AWS motif (associated with SET)	Contain SET, post-SET domain, PHD (plant homeodomain), PWWP (proline-tryptophan-tryptophan-proline), FYRN (F/Y rich N-terminus), and FYRC (F/Y rich C-terminus)	Contain SET domain and PHD domain	Contain SET domain, pre-SET domain, post-SET domain, and SRA (SET and RING finger associated domain)	Contain all other domains except SRA domain; some members are reported to possess a novel WIYLD domain similar to DNA-binding protein

Role	CLF helps in flowering time regulation; MEA is involved in seed development; SWN is required for flower development	SDG8 is involved in *FLC* expression which regulates flowering time; SDG4 is involved in proper pollen and stamen development	Involved in drought stress response; *atx1* mutant is hyposensitive to dehydration	Play role in cell cycle regulation	Mark inactive chromatin; gene silencing	Potential role in heterochromatic siRNA production machinery

Epigenetic Role	They have H3K27 methyltransferase activity	They carry out di- or trimethylation of H3K36	ATX1 and ATX2 are involved in H3K4me3 and H3K4me2 Function of ATX 3, 4, 5 is still not known	They carry out monomethylation of H3K27	H3K9 methylation; different SRA domains have preferential affinity for different cytosine methylation context (symmetric or asymmetric)	Function unclear yet; however, it resembles proteins involved in heterochromatin formation

**Table 2:** Types of Histone demethylases (HDMs).

Lysine specific demethylase 1 (KDM/LSD1)	Jumonji C domain containing proteins (JmjC)
Require flavin as cofactor	Require Fe(II) and α-ketoglutarate as cofactors

Remove methyl group from mono- and dimethylated lysines on histone tails.	Remove methylation from mono-, di-, and trimethylated lysines

Four KDM/LSD1 demethylases in *Arabidopsis*: Flowering locus D (FLD) LSD1 like (LDL1) LDL2 LDL3	Twenty one JmjC-domain proteins are reported in *Arabidopsis* which are classified into 5 subfamilies: KDM5/JARID1 group KDM4/JHDM3 group KDM3/JHDM2 group JMJD6 group JmjC-domain only

FLD, LDL1, and LDL2 are involved in flower induction in *Arabidopsis* through *flc* repression	KDM4/JHDM3 proteins along with ELF6/JMJ11 (early flowering 6) and REF/JMJ12 (relative of early flowering) control flowering time (Yu et al. 2008) [40], whereas KDM3/JHDM2 protein, for instance, IBM1/JMJ25 (increase in bonsai methylation), protects active genes from ectopic H3K9me2 and CNG DNA methylation [41]
